# The differential cancer growth associated with anaesthetics in a cancer xenograft model of mice: mechanisms and implications of postoperative cancer recurrence

**DOI:** 10.1007/s10565-022-09747-9

**Published:** 2022-08-12

**Authors:** Masae Iwasaki, Hailin Zhao, Cong Hu, Junichi Saito, Lingzhi Wu, Aislinn Sherwin, Masashi Ishikawa, Atsuhiro Sakamoto, Donal Buggy, Daqing Ma

**Affiliations:** 1 Division of Anaesthetics, Pain Medicine and Intensive Care, Department of Surgery and Cancer, Faculty of Medicine, Imperial College London, Chelsea & Westminster Hospital, 369 Fulham Rd, Chelsea, London, SW10 9NH UK; 2 Department of Anaesthesiology and Pain Medicine, Graduate School of Medicine, Nippon Medical School, Tokyo, Japan; 3 Department of Anesthesiology, Hirosaki University Graduate School of Medicine, Hirosaki, Aomori, Japan; 4 Anaesthesiology and Perioperative Medicine, Mater University Hospital, University College Dublin, Dublin, Ireland

**Keywords:** Anaesthetic effects, Propofol, Sevoflurane, Tumour progression

## Abstract

Anaesthetics may modify colorectal cancer cell biology which potentially affects long-term survival. This study aims to compare propofol and sevoflurane regarding with the direct anaesthetic effects on cancer malignancy and the indirect effects on host immunity in a cancer xenograft mode of mice. Cultured colon cancer cell (Caco-2) was injected subcutaneously to nude mice (day 1). Mice were exposed to either 1.5% sevoflurane for 1.5 h or propofol (20 μg g^−1^; ip injection) with or without 4 μg g^−1^ lipopolysaccharide (LPS; ip) from days 15 to 17, compared with those without anaesthetic exposure as controls. The clinical endpoints including tumour volumes over 70 mm^3^ were closely monitored up to day 28. Tumour samples from the other cohorts were collected on day 18 for PCR array, qRT-PCR, western blotting and immunofluorescent assessment. Propofol treatment reduced tumour size (mean ± SD; 23.0 ± 6.2mm^3^) when compared to sevoflurane (36.0 ± 0.3mm^3^) (*p* = 0.008) or control (23.6 ± 4.7mm^3^). Propofol decreased hypoxia inducible factor 1α (HIF1α), interleukin 1β (IL1β), and hepatocyte growth factor (HGF) gene expressions and increased tissue inhibitor of metalloproteinases 2 (TIMP-2) gene and protein expression in comparison to sevoflurane in the tumour tissue. LPS suppressed tumour growth in any conditions whilst increased TIMP-2 and anti-cancer neutrophil marker expressions and decreased macrophage marker expressions compared to those in the LPS-untreated groups. Our data indicated that sevoflurane increased cancer development when compared with propofol in vivo under non-surgical condition. Anaesthetics tested in this study did not alter the effects of LPS as an immune modulator in changing immunocyte phenotype and suppressing cancer development.

**Supplementary Information:**

The online version contains supplementary material available at 10.1007/s10565-022-09747-9.

## Introduction

Colorectal cancer is the third most common cancer, with approximately 1.8 million new cases each year globally. Despite improvements in patients’ survival with newer cytotoxic regimens and enormous investment in the development of cancer targeted pharmacological therapies over recent decades, surgical resection remains the most effective treatment for most solid organ cancer patients as it offers the best chance of cure. However, it has recently been recognised that surgery itself has a direct impact on tumour biology. Reports have focussed principally on how the surgical stress results in immunosuppression that may increase the risk of post-operative recurrence or metastatic spread. However, there are limited investigations into what the effects of anaesthetics administered during cancer surgeries have any influence on cancer surgical outcomes.

Growing evidence suggests a potential impact of anaesthetic technique and surgical inflammation on cancer recurrence following surgery (Tavare et al. 2012). In fact, retrospective clinical studies demonstrated that total intravenous anaesthesia with propofol is associated with a reduced risk of cancer recurrence and better long-term survival, when compared to inhalational anaesthesia with sevoflurane or desflurane (Forget et al. 2010; Wigmore et al. 2016). Pro-cancer factors, e.g. hypoxia inducible factors 1α (HIF1α) and hepatocyte growth factor (HGF) or anti-cancer factors like tissue inhibitor of metalloproteinase 2 (TIMP-2) have been associated with cancer aggressiveness or metastasis (Talks et al. 2000; Unwith et al. 2015) in experimental models of cancer progression^4^. There is increasing evidence suggesting that anaesthetics exert long lasting biological effects on the body cells as a whole due to strongly induced cellular signalling changes that continue long beyond the primary actions of anaesthetics and analgesics (Hovaguimian et al. 2020). Indeed, we have demonstrated the direct effects of anaesthetics on prostate (Huang et al. 2014), renal (Benzonana et al. 2013), ovarian (Iwasaki et al. 2016) and lung cancer cell biology (Hu et al. 2021) and tumour metastatic genes (Hu et al. 2021; Iwasaki et al. 2016).

In addition, previous studies also indicated that perioperative anti-inflammatory medication reduced cancer recurrence following surgery (Wang et al. 2018), whereas postoperative inflammation including abdominal sepsis worsens long-term survival in colorectal cancer patients (Breugom et al. 2016). While some studies suggest that anaesthetics may weaken anticancer immunity of the patients in vitro, the whole picture remains unclear (Iwasaki et al. 2015). Of those, it has been reported that lipopolysaccharide (LPS) intravenous or intraperitoneal injection shrunk the allograft and xenograft tumour volume (Kocijancic et al. 2017; Masuda et al. 2018).

In this regard, the interaction between metastasis-related mechanisms and host immune response under inflammation represents an important clinical aspect that needs to be taken into account, when considering anaesthetic regimens for cancer surgery and other potential anti-cancer therapeutics.

This study aims to investigate the effects of sevoflurane and propofol on cancer cell biology, and their respective effects on LPS-induced tumour immunity in a nude mice xenograft model. Our hypotheses are that propofol exposure, either alone or in combination with LPS injection, may induce inhibitory molecular changes in xenograft tumours, thereby potentially reducing tumour growth and improving clinical outcomes.

## Material and methods

### Ethics statement

All animal procedures were conducted in accordance with the United Kingdom Animals Scientific Procedures Act of 1986 and were approved by the Home Office, UK (Project approval no: 70/8496).

### Cell culture

Human authenticated colon cancer cell line, Caco-2, was cultured in DMEM medium with GlutaMAX™-I (Thermo Scientific, Loughborough, UK), containing 10% foetal bovine serum (Thermo Scientific, Loughborough) and 1% penicillin (Sigma-Aldrich, Dorset, UK) and maintained in a humidified incubator at 37 °C with a 5% CO_2_ atmosphere.

### Xenograft model with anaesthesia and LPS injection

Adult male BALB/C immunodeficient nude mice (Harlan, UK) were housed in temperature- and humidity-controlled cages in a pathogen-free facility at Chelsea and Westminster Campus, Imperial College London (UK). Caco-2 tumour cells (approximately 1 × 10^7^ cells) were suspended in 200 μl of RPMI1640 medium (Sigma-Aldrich) and injected subcutaneously into the mice (day 1). The tumour diameters in three axes and mice body weights were monitored on every other day. Tumour volumes were estimated as ellipsoids with radius in three axes (a, b and c) using the following formula:

$$V=4/3\pi abc$$

Two weeks after tumour cell implantation, mice were exposed to one of the following three conditions: air (control group; NC); 1.5% sevoflurane for 1.5 h (sevoflurane group; S) or 20 μg g^−1^ propofol intraperitoneal injection (propofol group; P) once a day, for three consecutive days (days 15–17, *n* = 6). The propofol dose was derived from our pilot study, and it caused light sedation without any cardiopulmonary suppression for 1.5 h. The experimental gas mixtures were O_2_ 1 l min^−1^ and air 2 l min^−1^ with or without anaesthetics. The F_*I*_O_2_ and anaesthetic concentration were measured with capnometer (Datex-Ohmeda, Stirling, UK). Lipopolysaccharide (LPS) groups received 4 μg g^−1^ LPS (Sigma-Aldrich) intraperitoneal injection once daily after full recovery from sevoflurane exposure or propofol injection for three consecutive days (days 15–17, *n* = 6). The sedation level was evaluated with mice grimace scale (Langford et al. 2010), which was done without stimulation. Mice were sacrificed on day 18 (*n* = 3 per group), and ex vivo tumour samples were harvested in RNAlater RNA stabilization reagent (Qiagen, West Sussex, UK) at − 20 °C until RNA experiments, in 4% paraformaldehyde at 4° C or kept at − 80 °C for further analysis. The clinical endpoints of the survival study in the other cohorts were assessed with general conditions including body weight loss more than 20% and tumour volume over 70mm^3^, and their survival were closely monitored up to day 28 (*n* = 3). The lung and liver were extracted from mice after reached the clinical end points (*n* = 6) and fixed in 4% paraformaldehyde at 4 °C for further analysis.

### RNA extraction, PCR array and qRT-PCR

Total RNA was extracted from ex vivo tumour samples using the RNeasy mini kit® and QIAshredder (Qiagen) according to the manufacturer’s instructions. RNA quantity and quality were assessed using a BioPhotometer (Eppendorf, Stevenage, UK). Samples with an A260:A280 ratio > 1.8 were considered to be sufficient quality for further analysis.

PCR array analysis was performed using a RT^2^ Profiler™ PCR Array Human Tumour Metastasis (Qiagen). The RT^2^ First Strand Kit (Qiagen) was used to produce complementary DNA (cDNA) from 1 μg of total RNA. cDNA samples were mixed with RT^2^ SYBR Green ROX FAST Mastermix (Qiagen) before loading into each well of the PCR array. PCR array plates were processed and analysed with Rotor Gene Q system (Qiagen). mRNA expression relative to Glyceraldehyde-3-phosphate dehydrogenase (GAPDH) mRNA was determined using the comparative 2^−ΔΔCT^ (with supplement software provided by Qiagen) method, and these values were subsequently converted into cluster gram to show relative levels of mRNA expression.

Some genes from above array experiments and their expression were quantified using the Rotor-Gene Q system (Qiagen) in the presence of SYBR green (Qiagen). cDNA was mixed with mastermix, and forward and reverse primers of each probe. Paired oligonucleotide primers were designed for tissue inhibitor of metalloproteinases-2 (TIMP-2), hypoxia-inducible factor 1α (HIF1α), epithelial cadherin (E-cadherin), interleukin 1β (IL1β), vascular endothelial growth factor A (VEGFA), tumour necrosis factor α (TNFα), CD68, Arginase1, inducible NO synthase (iNOS), nuclear factor κ-light-chain-enhancer of activated B cells (NFκB), C–C chemokine receptor type 2 (CCR2), myeloperoxidase (MPO) and GAPDH using Primer Designer (Scientific and Educational Software, Durham, USA) against the sequence downloaded from GenBank and were supplied by Invitrogen; the RT^2^ qPCR Primer Assay for hepatocyte growth factor (HGF) was supplied by Qiagen. The primer sequences, *r*^2^ values and efficiencies are summarized in the supplemental Table 1. All mRNA data were expressed relative to the endogenous control gene, GAPDH.

### Immunofluorescent staining

Ex vivo tumour samples preserved in 4% paraformaldehyde were then dehydrated in 40% sucrose solution at 4 °C. Tumour tissues were embedded in o.c.t compound (VWR, Leicestershire, UK) and cryosectioned into 25-μm-thick sections. Frozen sections were blocked in 5% normal donkey serum at room temperature for 1 h and then incubated overnight at 4 °C with one of the primary antibodies: mouse monoclonal HGF (Santa Cruz Biotechnology, TX, USA), mouse monoclonal TIMP-2 (Santa Cruz Biotechnology), mouse monoclonal CD68 antibody (Santa Cruz Biotechnology), rat monoclonal Ly6g antibody (Abcam plc, Cambridge, UK), rabbit monoclonal CD11c antibody (Cell Signalling technology, London, UK) or mouse CD54 antibody (Novus biologicals, Abingdon, UK). Slides were then incubated the following day with Alexa flour 568-conjugated (ThermoFisher scientific) or Alexa flour 488-conjugated (Abcam plc) secondary antibody incubation. Slides were counterstained with Vectashield mounting medium containing DAPI (Millipore, Watfield, UK). Slides were viewed under Olympus BX4 microscope (Olympus, Hamburg, Germany).

### Histological study

Ex vivo liver and lung tissue samples preserved in 4% paraformaldehyde were dehydrated with xylene and ethanol overnight. After being fixed with paraffin, liver and lung samples were sectioned into 5-μm-thick sections and stained with H&E to search for cancer metastasis.

### Western blotting

Western blotting was done using our established protocol (Iwasaki et al. 2016), and 40-μg protein samples were loaded to each well. Briefly, after electrophoresis and transfer onto a polyvinylidenedifluoride (PVDF) membrane using the iBlot® 2 Dry Blotting System (ThermoFisher scientific), membranes were blocked with 5% non-fat powdered milk in Tris-buffered saline with Tween (TBS-T) for 1 h at room temperature, and then incubated overnight at 4 °C with anti-TIMP-2 mouse primary antibody (Santa Cruz Biotechnology), anti-HIF1α (Novus biologicals), anti-E-cadherin (Santa Cruz Biotechnology) or anti-GAPDH mouse antibody (Millipore), followed by horseradish peroxidase (HRP)-linked anti-rabbit or anti-mouse (Cell Signaling technology) secondary antibody for 1 h. Protein bands were visualised using the enhanced chemiluminescence (ECL) system (Santa Cruz Biotechnology) and the Syngene GeneSnap software (Syngene, Cambridge, UK). Western blotting band quantification was conducted with software ImageJ (https://imagej.nih.gov/ij/) relative to GAPDH expression.

### Statistical analysis

All numerical data are presented as scatter dot plot and mean ± SD. A group size of *n* = 6 is needed to show a 30% change with 80% power at 5% significance at day 18. One-way ANOVA analysis followed by post hoc Tukey–Kramer’s test were applied for statistical analysis using Prism ver 5.0 (GraphPad Software, Inc., CA, USA), unless otherwise specified. For analysis of the array results (*n* = 3), the false discovery rate was used, set at 0.1 using the program QVALUE 2.0 (http:// genomics.princeton.edu/storeylab/qvalue/). Regarding biochemical data within and between groups, comparisons are made using factorial two-way ANOVA with post hoc Tukey–Kramer test. Kaplan–Meier survival analysis were used for survival with clinical endpoints (*n* = 6, up to day 28). In all experiments, an *α* of 0.05 is used for establishing significance.

## Results

### Propofol treatment reduced colon tumour size while sevoflurane promoted tumour growth

**Fig. 1:**
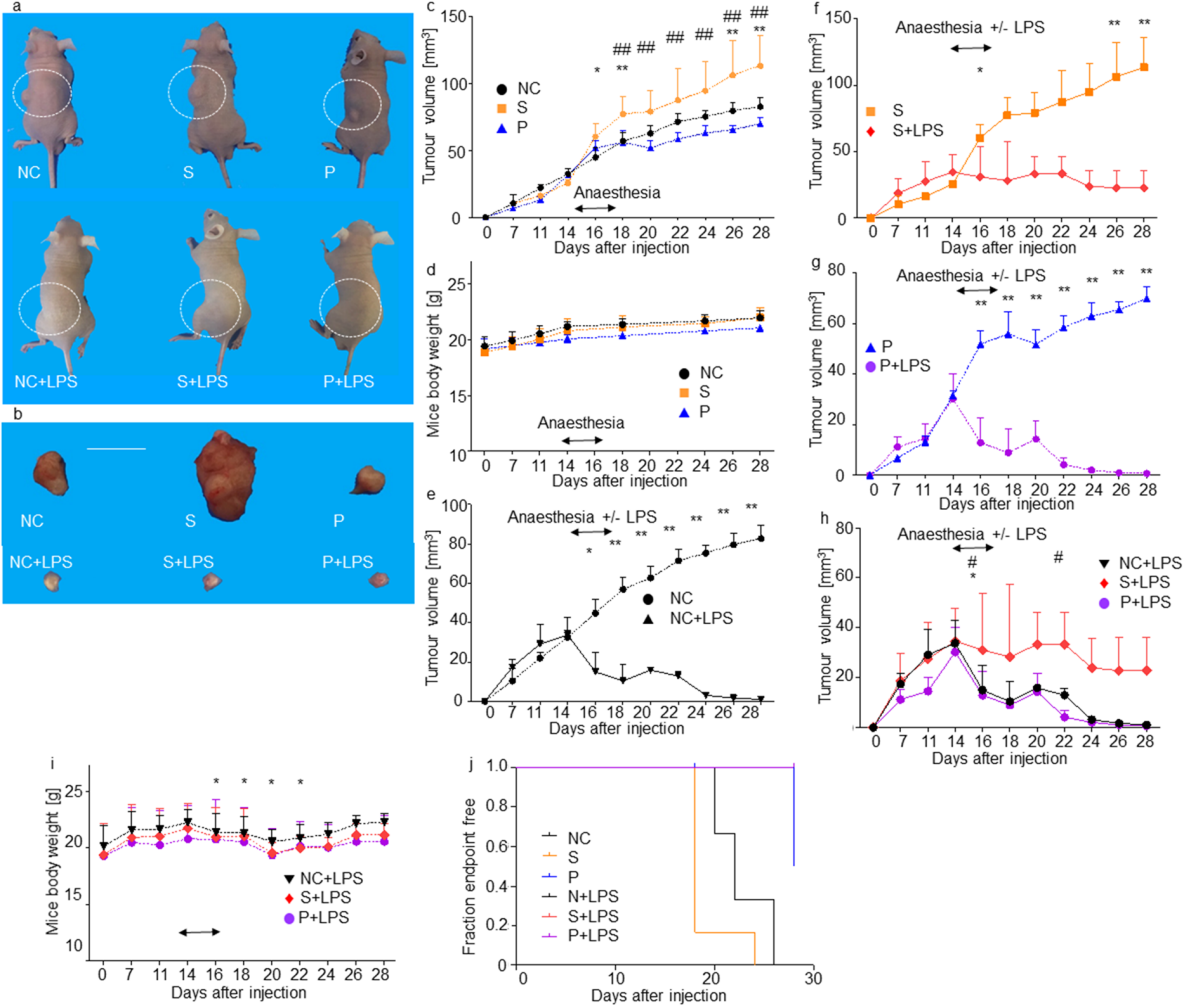
Tumour volume transition after sevoflurane and propofol exposure with or without LPS injection. **a** The comparison of general appearance of xenograft mice on day 18, control (left), 1.5% sevoflurane (middle) and 20 mg kg^−1^ propofol (right), without LPS injection (upper) or with LPS injection (lower). The subcutaneous tumours were circled; **b** The comparison of ex vivo Caco-2 tumours after 1.5-h anaesthesia exposure over three consecutive days, control (left), 1.5% sevoflurane (middle) and 20 mg kg^−1^ propofol (right), without LPS injection (upper) or with LPS injection (lower) (*n* = 6, scale bar = 5 mm); **c** The comparison of tumour volume after sevoflurane and propofol exposure with control group; **d** The mice body weight transition in LPS untreated groups; **e** The comparison of tumour volume with control with or without LPS injection groups; **f** The comparison of tumour volume with sevoflurane with or without LPS injection groups; **g** The comparison of tumour volume with propofol with or without LPS injection groups; **h** The comparison of tumour volume after sevoflurane or propofol exposure with LPS injection with control with LPS injection group, **i** The mice body weight transition within LPS-treated groups; **j** Clinical endpoint free population fraction of xenograft mice up to day 28. (tumour volume (mm.^3^), body weight (g), *n* = 6 up to day 28, One-way ANOVA with Tukey–Kramer, compared to each control group, *p < 0.05, **p < 0.01, comparison between S (sevoflurane) and P (propofol) or LPS-treated groups compared with the respective non-LPS S and P groups, #*p* < 0.05, ##*p* < 0.01)

In order to ascertain if there are any different effects of propofol and sevoflurane on cancer growth, we have closely monitored xenograft tumour volumes and mice appearance after general anaesthesia of clinical dose and for duration up to day 28. All anaesthesia levels were evaluated as ‘light anaesthesia’, thus no additional propofol injection was done. Amongst LPS untreated groups, tumour mass increased after sevoflurane exposure than the two other groups exposure on day 18 ((mean ± SD, mm^3^, *n* = 6); S 36.0 ± 10.3 vs NC 23.6 ± 4.7 vs P 23.0 ± 6.2; *p* = 0.008) (Fig. 1a and b). The ex vivo tumour size of the S group was the biggest among all six groups after day 16 to day 28 (Fig. 1c). No body weight loss was found among the groups without LPS injection (Fig. 1d). The significant decrease of tumour size was noted after LPS treatment in the NC + LPS (Fig. 1e), the S + LPS (Fig. 1f) and the P + LPS groups (Fig. 1g) after day 16 up to day 28 (Fig. 1h), compared with the respective LPS untreated groups (*p* < 0.01). Body weight loss was observed only in the LPS-treated groups from day 18 to day 24 compared to the LPS-untreated groups (Fig. 1i). All mice did not reach to any clinical endpoints in the LPS groups up to day 28. There was a significant difference in endpoint-free population on day 28, between the NC, S, P and all the LPS groups (NC 50% vs S 0% vs P 80% vs NC + LPS 100% vs S + LPS 100% vs P + LPS 100%, *p* < 0.01, Fig. 1j). There was a significant difference in the clinical endpoint-free duration (NC 22 days, S 18 days, P 28 days, NC + LPS undetermined, S + LPS undetermined, P + LPS undetermined, *n* = 6; *p* = 0.017).

### Propofol treatment modified fewer metastasis-related genes than sevoflurane exposure and upregulated TIMP-2 expression

**Fig. 2:**
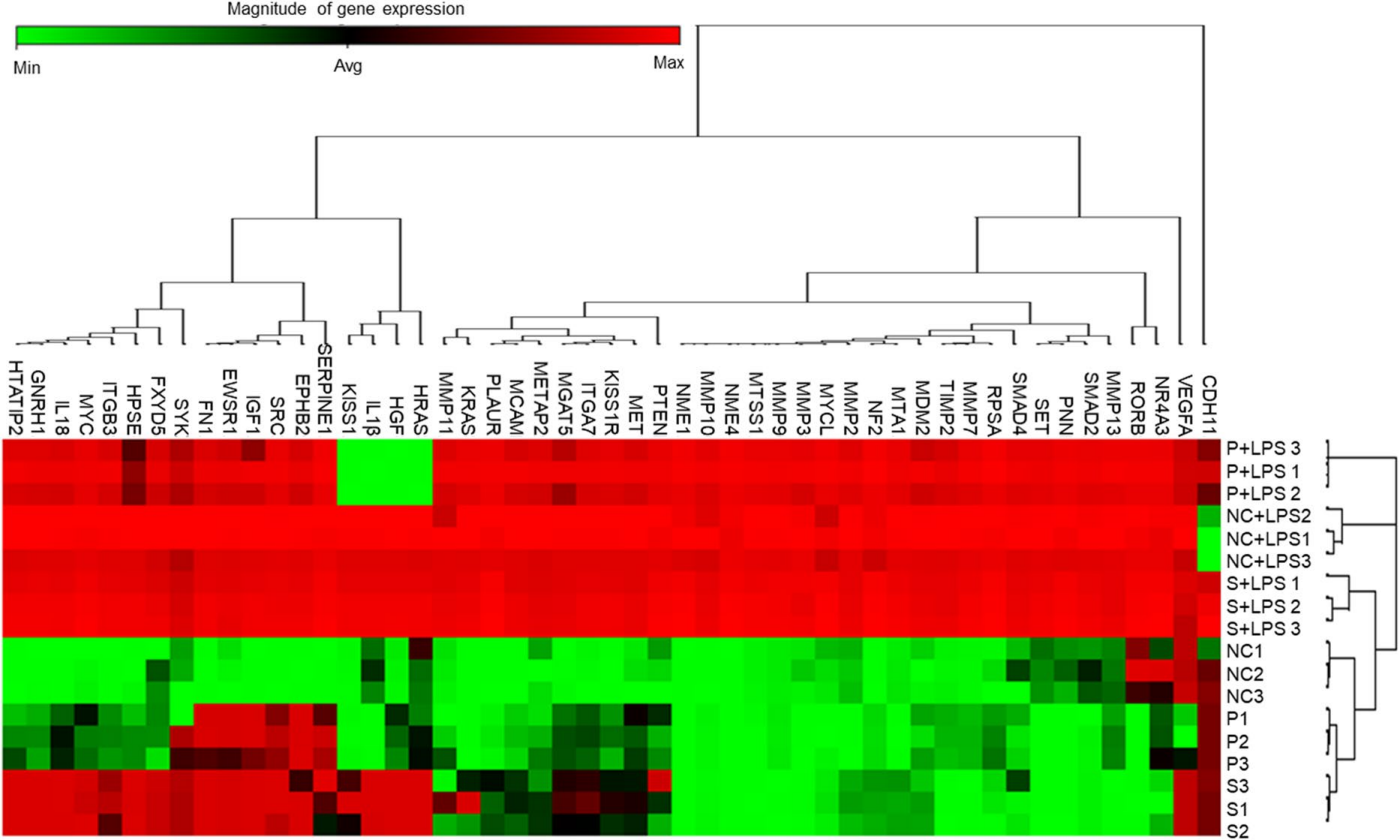
Sevoflurane, propofol and LPS injection modulated expressions of cancer metastasis–related genes in xenograft samples. Mice were treated with air (NC) or 1.5% sevoflurane (S) and or 20 mg kg^−1^ propofol (P) for 1.5 h with or without following 4ug g.^−1^ LPS injection, for three consecutive days. Tumour samples were harvested for tumour metastasis PCR array 24 h after the third exposure. Unsupervised hierarchical cluster analysis with Euclidean distance was used. All data is relative to endogenous control, GAPDH. Red and green colours indicate relatively high and low expression, respectively. (*n* = 3, *p < 0.05, one-way ANOVA followed by Tukey–Kramer)

In order to explore the gene expression pattern in xenograft tumours, we have carried out PCR array about cancer metastasis–related genes with validation by qRT-PCR, western blotting and immunofluorescence staining. The cluster analysis of PCR array showed each anaesthetic treatment changed cancer metastasis–related gene expressions in its own pattern (Fig. 2). Out of 84 cancer metastasis–related genes, 19 were upregulated by sevoflurane exposure, but only 2 (TIMP-2 and Cadherin-11; CDH11) were upregulated by propofol exposure relative to the NC (*n* = 3, Supplemental Table 2). When comparing the S and P groups, twelve genes were increased, including HGF, TIMP-2, VEGFA and IL1β. When comparing the LPS-treated groups vs LPS-untreated groups, fifty gene expressions changed significantly (Supplemental Table 2). Among the LPS-treated groups, six gene expressions of HGF, IL1β, VEGFA, V-Ha-ras Harvey rat sarcoma viral oncogene homolog (HRAS), KiSS-1 metastasis-suppressor (KISS1) and CDH11 were increased. The PCR array results were further validated with qRT-PCR of HGF, TIMP-2, IL1β and VEGFA, western blotting or immunofluorescent staining.

### Sevoflurane treatment upregulated gene and protein expression of HGF, HIF1α, IL1β and TNFα, and downregulated E-cadherin expression when compared to propofol

**Fig. 3:**
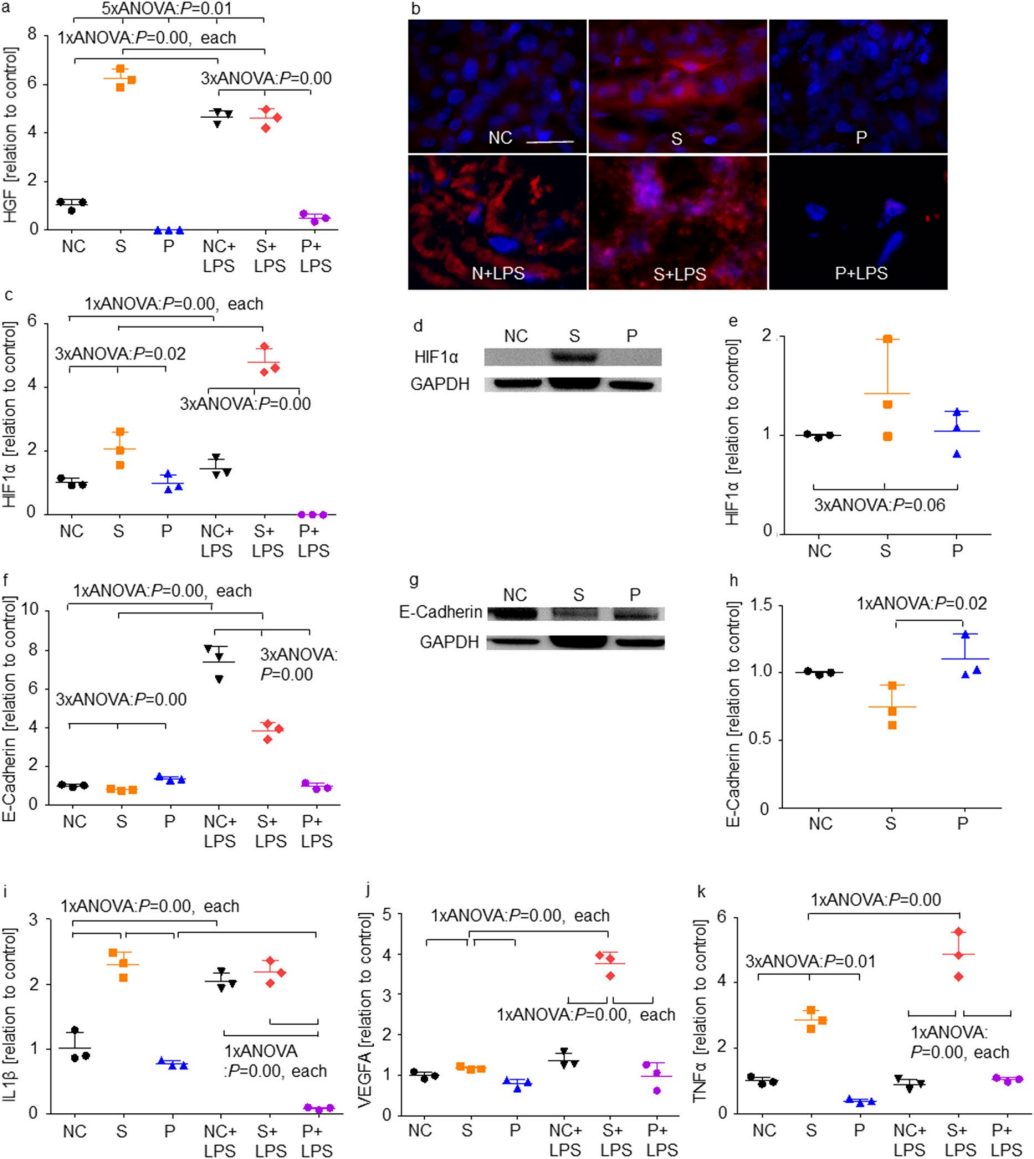
The expression changes of cancer malignancy markers after sevoflurane, propofol and LPS exposure. **a** HGF (a proliferative marker) expressions evaluated with qRT-PCR compared to control group; **b** Immunofluorescence staining, HGF (red), a proliferative marker, in control (left), sevoflurane (middle) and propofol-treated (right) with (upper) or without LPS treatment (lower) Caco-2 ex vivo samples, counterstained with DAPI (blue); × 20 magnification, scale bar = 20 μm; **c** HIF1α (a cancer malignancy marker) expressions evaluated with qRT-PCR; **d** The representative image of western blotting analysis for HIF1α among no LPS treatment groups; **e** The quantification of western blotting analysis for HIF1α among no LPS treatment groups; **f** E-cadherin (a migration marker) expressions evaluated with qRT-PCR; **g** The representative image of western blotting analysis for E-cadherin among no LPS treatment groups; **h** The quantification of western blotting analysis for E-cadherin among no LPS treatment groups; **i** IL1β (an inflammation marker) expressions evaluated with qRT-PCR; **j** VEGFA (an angiogenesis marker) expressions evaluated with qRT-PCR; **k** TNFα (an inflammation marker) expressions evaluated with qRT-PCR. Data showed as dot plots and mean ± SD (*n* = 3); *p < 0.05, **p < 0.01, one-way ANOVA followed by Tukey–Kramer compared with the NC group. NC: naïve control; S: sevoflurane; P: propofol; LPS: lipopolysaccharides; HGF: hepatocyte growth factor; HIF1α: hypoxia-inducible factor 1α; IL1β: interleukin 1β; VEGFA: vascular endothelial growth factor A; TNFα: tumour necrosis factor α

In order to investigate the roles of sevoflurane and propofol exposure, we have investigated changes of the inflammatory cytokines and cancer malignancy markers after general anaesthesia. Figure 3a-k showed results of qRT-PCR, western blotting analysis and immunofluorescence staining of the inflammatory cytokines and cancer malignancy markers. The numeral data of qRT-PCR was summarized in the supplemental Table 3. Both the gene and protein HGF was expressed in a high level in the S group (Fig. 3a  and b). With LPS treatment, HGF expression was increased in the N + LPS and S + LPS groups, not in the P + LPS group with qRT-PCR (Fig. 3a) and immunofluorescent staining (Fig. 3b). HIF1α expression was increased in the S and S + LPS groups significantly but downregulated in the P + LPS group (Fig. 3c, d and e). E-cadherin gene expression was upregulated in the NC + LPS and S + LPS groups (Fig. 3f). Among LPS groups, the S + LPS and P + LPS groups showed lower E-cadherin expression than the NC + LPS group. Protein assay showed lower E-cadherin expression in the S group than P group (Fig. 3g and h). IL1β gene expression was upregulated in the S group than the NC and P groups, and in the NC + LPS and P + LPS groups (Fig. 3i). The P + LPS group showed lower IL1β expression than the P group. VEGFA gene expression was upregulated only in the S + LPS group (Fig. 3j) that concurs with PCR array results. TNFα gene expression was upregulated in the S group but downregulated in the P group compared with the NC group (Fig. 3k). With LPS injection, the NC + LPS group showed lower TNFα expression than the NC group, but the S + LPS and P + LPS groups showed higher expression than the S and P groups, respectively.

### Propofol exposure increased TIMP-2 expression whereas LPS addition induced high MPO and CCR2 expression

**Fig. 4:**
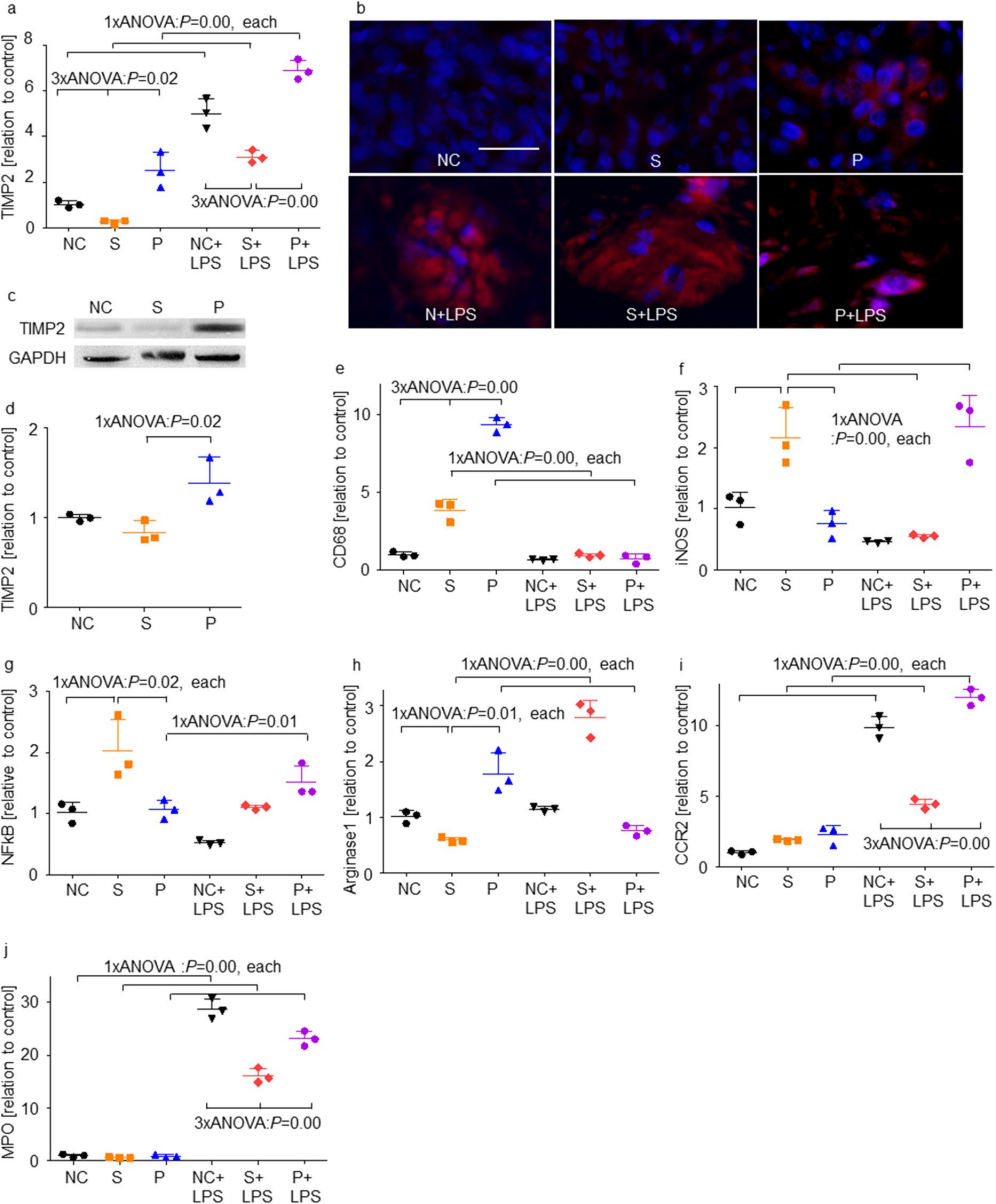
The anti-cancer factors and immune phenotype markers changed after sevoflurane, propofol and LPS exposure. **A** TIMP-2 (an MMPs regulator) expression evaluated with qRT-PCR compared to control group; **B** Immunofluorescence staining, TIMP-2 (red), a MMPs regulator, in control (left), sevoflurane (middle) and propofol-treated (right) with (upper) or without LPS treatment (lower) Caco-2 ex vivo samples, counterstained with DAPI (blue); × 20 magnification, scale bar = 20 μm; **C** Western blotting analysis for TIMP-2 among no LPS treatment groups; **D** The quantification of western blotting analysis for TIMP-2 among no LPS treatment groups; **E** CD68 (a macrophage marker) expression evaluated with qRT-PCR; **F** iNOS (an M1 macrophage marker) expression evaluated with qRT-PCR; **G** NFκB-p65 (an M1 macrophage marker, LPS target) expression evaluated with qRT-PCR; **H** Arginase-1 (an M2 macrophage marker); **I** CCR2 (a neutrophil and monocyte marker) expression evaluated with qRT-PCR; **J** MPO (a neutrophil marker) expression evaluated with qRT-PCR. Data showed as dot plots and mean ± SD (*n* = 3). *p < 0.05, **p < 0.01, One-way ANOVA followed by Tukey–Kramer compared with NC group. NC: naïve control; S: sevoflurane; P: propofol; LPS: lipopolysaccharides; TIMP-2: tissue inhibitor of metalloproteinases 2; iNOS: inducible NO synthase; NFκB: nuclear factor κ-light-chain-enhancer of activated B cells; CCR2: C–C chemokine receptor type 2; MPO: myeloperoxidase

We then explored the effects of sevoflurane and propofol on anti-tumour factors and phenotype changes of macrophages and neutrophils. TIMP-2 gene expression was increased after propofol exposure, or LPS treatment with any anaesthetics given, compared with the NC group (Fig. 4a). The P group showed a high TIMP-2 expression in the cytoplasm (Fig. 4b), and higher protein expression than in those of the NC and S group (Fig. 4c and d). CD68 gene expression was significantly increased only in the S and P groups; no increase was observed in all the LPS treated groups (Fig. 4e). iNOS and NFκB expressions were increased in the S group compared with the NC group, and such upregulation was suppressed with LPS treatment (Fig. 4f and g). Amongst LPS-treated groups, P + LPS group showed increases in iNOS and NFκB expression. Arginase 1 expression increased in the P group compared with the NC, but its expression in the P + LPS group was suppressed to the NC level (Fig. 4h). No changes were observed in the S group, whereas S + LPS group showed an increase of arginase 1 expression. qRT-PCR data of CCR2 and MPO showed a similar tendency, with increased expression in the LPS-treated group compared with the NC group (Fig. 4i and j). Amongst the LPS groups, S + LPS group showed a decrease of CCR2 and MPO expression than those in the NC + LPS and P + LPS groups.

### *Propofol and sevoflurane exposure increased CD68 positive cells in *ex vivo* tumour whereas LPS addition increased Ly6g positive cells*

**Fig. 5:**
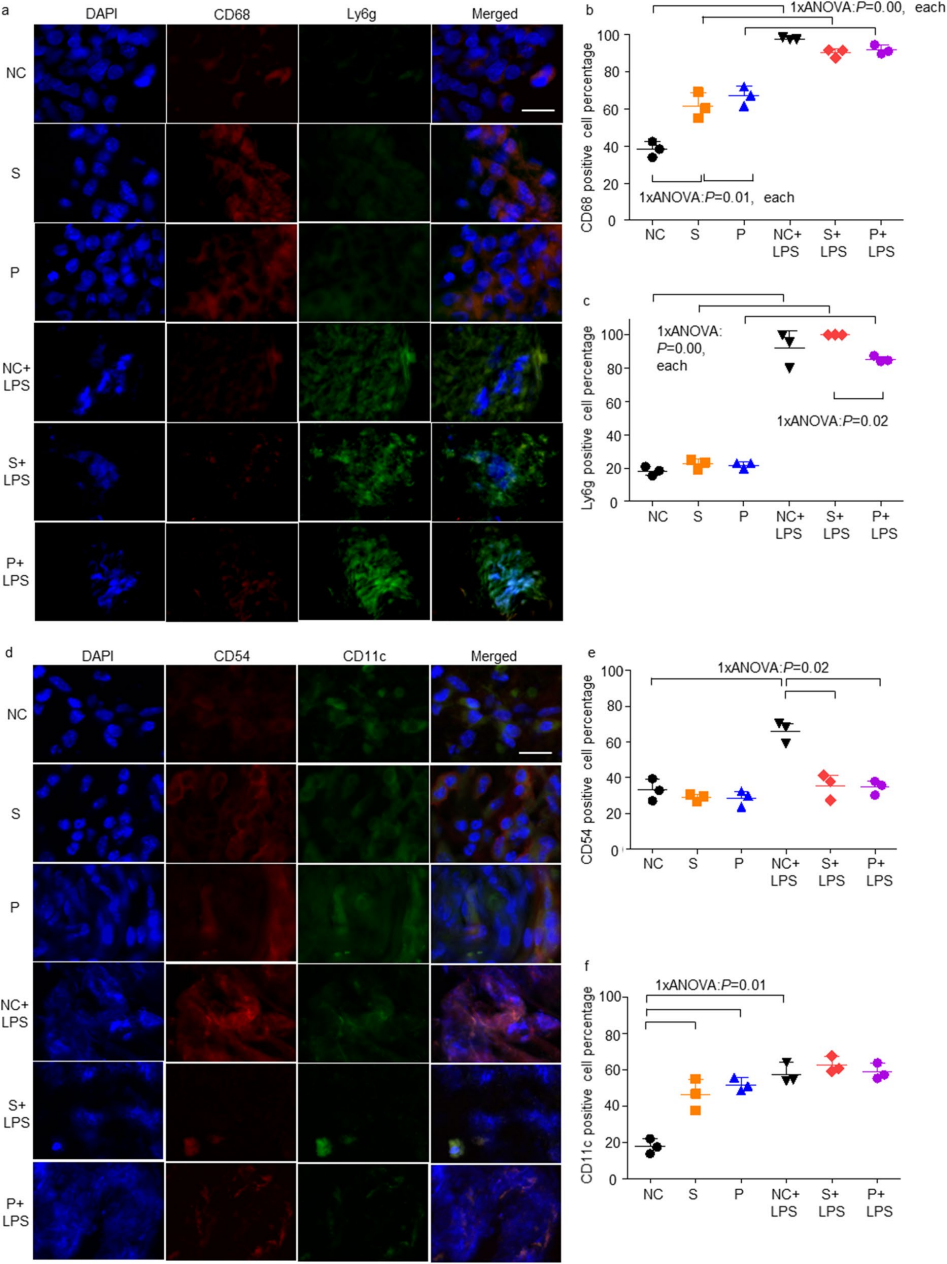
The changes of the surface marker expressions for pan-macrophage, cytotoxic neutrophils, anti-cancer phenotypes of macrophages and neutrophils after sevoflurane, propofol and LPS exposure. **A** Immunofluorescence staining, CD68 (red), a macrophage marker, in control (upper), sevoflurane (middle) and propofol-treated (lower) with (upper) or without LPS treatment (lower) Caco-2 ex vivo samples, counterstained with Ly6g, a neutrophil marker (green) and DAPI (blue); × 20 magnification, scale bar = 20 μm; **B** CD68 positive cell percentage in the immunofluorescence staining images from ex vivo tumour sections at day 18; **C** Ly6g positive cell percentage in the immunofluorescence staining images from ex vivo tumour sections at day 18; **D** Immunofluorescence staining, CD54 (red), an N1 neutrophil marker, in control (upper), sevoflurane (middle) and propofol-treated (lower) with (upper) or without LPS treatment (lower) Caco-2 ex vivo samples, counterstained with CD11c, an M1 macrophage marker (green) and DAPI (blue); × 20 magnification, scale bar = 20 μm; **E** CD54 positive cell percentage in the immunofluorescence staining images from ex vivo tumour sections at day 18; **F** CD11c positive cell percentage in the immunofluorescence staining images from ex vivo tumour sections at day 18. NC: naïve control; S: sevoflurane; P: propofol; LPS: lipopolysaccharides; Ly6g: lymphocyte antigen 6 complex locus G6D

To reveal the anaesthetic effects on immunity, the expressions of macrophages and neutrophils markers were determined. Number of CD68-positive cells increased in the S and P groups compared to the NC group. On the other hand, Ly6g positive cells increased in the NC + LPS, S + LPS and P + LPS groups. The cell numbers were decreased in all the LPS groups (Fig. 5a). Percentages of CD68 or Ly6g positive cells were shown in Fig. 5b and c.

### Sevoflurane and propofol exposure increased N1 markers which decreased by LPS addition

To reveal the anaesthetic effects on anti-cancer phenotypes, we investigated the marker expressions for anti-cancer macrophages or neutrophils in ex vivo tumours. Among the LPS-untreated groups, CD54-expressing cells were increased in the S and P groups compared to the NC group. CD54 positive cells were increased in the NC + LPS group compared to the NC group, but decreased in the S + LPS and P + LPS groups compared with the S and P group, respectively. On the other hand, CD11c positive cells were decreased in the NC + LPS, S + LPS and P + LPS groups compared to the LPS-untreated groups. The positive cell numbers were decreased in all the LPS groups (Fig. 5d). Percentages of CD54 or CD11c positive cells were shown in Fig. 5e and f. There was no sign of cancer metastasis in mice liver and lung sections (Supplemental Fig. 1a and b). In mice lung, there were heavy cell infiltrations along the alveolar walls in the S, NC + LPS, S + LPS and P + LPS groups compared with each control group, but the tissue general structure was maintained (Supplemental Fig. 1b).

## Discussion

This study investigated the respective effects of inhalational anaesthetic sevoflurane and intravenous anaesthetic propofol (with or without LPS challenge) on the metastatic potential and molecular changes of Caco-2 cells in a non-surgical nude mice xenograft model. Unlike propofol, sevoflurane exposure resulted in bigger tumour size, shorter survival time (assessed with the clinical endpoints, including body weight loss > 20% and tumour volume > 70 mm^3^), higher HGF, HIF1α, IL1β and TNFα expressions, and lower E-cadherin and TIMP-2 expressions. LPS challenge eliminated sevoflurane-induced cancer growth, increased MPO and CCR2 expression and neutrophil infiltration, and reduced pan macrophage infiltration. The PCR array analysis showed that more metastasis-related genes were upregulated after sevoflurane than propofol exposure, and that LPS injection altered most of these gene expressions.

There are accumulating data suggesting that HIF1α and HGF may act as pro-tumour factor while TIMP-2 is an anti-cancer regulator. Our previous studies showed that inhalational anaesthetics upregulated HIF1α and promoted cell migration and proliferation in several cancer cell lines (Benzonana et al. 2013; Huang et al. 2014; Luo et al. 2015). Hepatocyte growth factor (HGF) is known as a pro-cancer promotor in cancer development (Michieli et al. 1999), invasion (Date et al. 1998), metastasis and drug resistance of colorectal cancer (Liska et al. 2011; Luraghi et al. 2014) through modulating mesenchymal epithelial transition (cMET) (Michieli et al. 1999). In vitro, interference of the HGF-cMET pathway inhibited tumour growth and migration (Zhang et al. 2019). On the other hand, TIMP-2, an MMPs (matrix metalloproteinase) regulator, inhibited cell proliferation, tumour growth, angiogenesis (Seo et al. 2003), cancer cell invasion (Stetler-Stevenson 2008) and cancer metastasis (Guan et al. 2017). As demonstrated in vitro and in vivo, TIMP-2 treatment to cancer cells prevented cell migration (Kim et al. 2012; Zhang et al. 2013), resulting in reduced tumour growth and less angiogenesis (Seo et al. 2003). It was reported that TIMP-2 interacted with pro-cancer factors, like HIF1α and VEGF-A (Lee et al. 2010). Specifically, a regulatory feedback circuit between HIF1α and TIMP-2 via miR-210 may exist (Kai et al. 2016). The present study showed that propofol decreased HIF1α and HGF expressions, and increased TIMP-2 expression compared with sevoflurane. Taken together, propofol exposure may lower cancer malignancy potential by various mechanisms such as inhibition of the endogenous pro-cancer cell signalling or pathways.

The present study also demonstrated that LPS injection after general anaesthetic exposure induced tumour volume reduction, high MPO and CCR2 expression, more neutrophil infiltration, and less M1 and pan macrophage infiltration. It is widely recognized that macrophages and neutrophils can change their phenotype polarizations depending on the tumour microenvironment, namely anti-tumour (M1 and N1) or pro-cancer (M2 and N2), respectively. N1 has more cytotoxicity and was seen in the surrounding region of the developing tumours within 1 week after implantation, compared to the established tumours (Mishalian et al. 2013). M2 and N2 are pro-tumour phenotypes and promote tumour growth mainly in established tumours (Granot and Fridlender 2015). Several previous studies showed that LPS injection to nude mice changed the immune cell phenotypes. For example, LPS ip injection to the xenograft mice modulated the macrophage phenotype polarization in blood and spleen, and changed M1 or M2 macrophage infiltration into the xenograft tumour and spleen (Masuda et al. 2018), and increased CD11c-Gr-1 macrophage infiltration in the lung (Rega et al. 2013). Furthermore, LPS injection to nude mice without tumour burden induced Gr-1 + CD11b + cell surge and CD11b or CD80 positive cell infiltration into spleen. LPS injection to xenograft mice increased tumour specific CD8 + T cells which prevented tumour growth (Kocijancic et al. 2017). Our data showed that general anaesthesia itself can modulate the monocyte and neutrophil phenotypes and infiltration into xenograft tumours, but LPS injection following general anaesthesia induced their anti-cancer phenotype changes, possibly reducing the metastatic potential of cancer.

Clinical pathological studies found high levels of cellular signal changes, e.g. HIF1α(Talks et al. 2000), HGF (Huang et al. 2017) and TIMP-2 (Kikuchi et al. 2000), in the majority of primary tumours and their metastases. High expression of HIF1α in cancer tissue is believed to associate with high therapeutic resistance, poor survival and more metastasis in patients (Talks et al. 2000). High HGF expression in cancer tissue or serum was found to correlate with advanced cancer stage and poor outcome (Huang et al. 2017; Toiyama et al. 2009). High MET expression in cancer tissue was considered to correlate to advanced tumour status, lymph node metastasis and poor survival (Takeuchi et al. 2003; Zeng et al. 2008). Molecular targeted therapy against HGF-cMET pathway has shown effectiveness in some clinical trials (Catenacci et al. 2017; Van Cutsem et al. 2019). In colorectal cancer patients, higher MMP:TIMP-2 ratio or reduced TIMP-2 expression in serum or tissue is known to be directly correlated with increased colorectal tumour invasion (Kikuchi et al. 2000) and poor prognosis (Park et al. 2011; Zhang et al. 2013). Higher TIMP-2 expression was related to the better overall survival (Kikuchi et al. 2000). In terms of macrophage and neutrophil phenotypes, previous studies indicated that MPO positive neutrophil infiltration predicts better survival and response to 5-FU-based chemotherapy in colorectal cancer (Berry et al. 2017; Galdiero et al. 2016). In contrast, macrophage infiltration into tumours predisposes chemoresistance due to their immunomodulating interaction (Yin et al. 2017). Clearly, those molecular entities discussed above, and their associated cellular signalling changes play particularly important roles on cancer development during perioperative period. If any interventions or medications (including anaesthetics) can modulate those molecules and influence cancer microenvironment and immunity of cancer patients, then surgical outcome may be changed. Indeed, the use of propofol in the total intravenous anaesthesia (TIVA) was found to be preferable in terms of postoperative metastasis and outcome (Wigmore et al. 2016; Wu et al. 2018). Clinical studies also indicated that perioperative anti-inflammatory medication reduced cancer recurrence following surgery (Forget et al. 2010; Huang et al. 2018). However, this area of research is just beginning, and more studies are urgently needed.

Our work is not without limitations. First, the anaesthetic doses and durations applied in our study were not directly close to clinical settings, but they are within the clinical dose/concentration range, and hence, our work may be still clinically relevant. Second, surgery was not used in the model; How surgical insults influence the implanted cancer development and progression remains elusive. Third, the xenograft model was used in our study which may be very different from the model derived from animal cancer cells in which tumour cell proliferation, migration, invasion and angiogenesis and underlying mechanisms can be better studied. Therefore, further study is needed to validate the current findings. Lastly, LPS was used in our study as an immunomodulator; however, itself is very toxic to cancer cells. Whether there were any “direct killing” effects in addition to itself “immunotherapy” in attenuating cancer xenograft development is unknown. Nevertheless, our work is a proof-of-concept study, and its translational value is worth pursuing further.

## Conclusion

This in vivo study showed that, in contrast to propofol, sevoflurane exposure resulted in bigger ex vivo tumour size, shorter survival time, higher HGF and HIF1α expression, and lower TIMP-2 expression than propofol injection. Anaesthetics did not change the immunomodulating ability of LPS, which enhances the anti-tumour phenotypes of monocytes and neutrophils. The clinical implications of this study warrant further study.

## Supplementary Information

Below is the link to the electronic supplementary material.

Supplementary file1 (DOCX 533 KB)

## Data Availability

Data is available on request from the authors.

## Abbreviations

LPS: Lipopolysaccharides
HGF: Hepatocyte growth factor
HIF1α: Hypoxia-inducible factor 1α
IL1β: Interleukin 1β
VEGFA: Vascular endothelial growth factor A
TNFα: Tumour necrosis factor α
