# Supplementary material for: The differential cancer growth associated with anaesthetics in a cancer xenograft model of mice: mechanisms and implications of postoperative cancer recurrence
Source: Cell Biol Toxicol. 2022 Aug 12;39(4):1561–75. doi: 10.1007/s10565-022-09747-9 (PMC10425502; doi:10.1007/s10565-022-09747-9)
Supplement: Supplementary file 1 — Supplementary file1 (DOCX 533 KB) [file 10565_2022_9747_MOESM1_ESM.docx]

**Supplemental table 1**. The primer list for qRT-PCR

**Supplemental table 2.** The array analysis of metastasis related genes after general anaesthesia (S=sevoflurane, P=propofol) with or without lipopolysaccharides (LPS) injection compared with the control group (NC).

**Supplemental table 2(a).** Genes which were upregulated in the sevoflurane and propofol groups in array analysis

**Supplemental table 2(b).** Genes which were upregulated only in the propofol group in array analysis

**Supplemental table 2(c).** Genes which were upregulated only in the sevoflurane group in array analysis

**Supplemental table 2(d).** Genes which were upregulated in the sevoflurane group but downregulated in the propofol group in array analysis

**Supplemental table 2(e).** Genes which were downregulated in the sevoflurane and propofol groups, upregulated with lipopolysaccharides (LPS) injection in array analysis

**Supplemental table 2(f).** Genes which were downregulated in the sevoflurane group but upregulated in the propofol and all the lipopolysaccharides (LPS) group in array analysis

**Supplemental table 2(g).** Genes which were downregulated only in the sevoflurane group, upregulated with lipopolysaccharides (LPS) injection in array analysis

**Supplemental table 2(h).** Genes which were downregulated only in the propofol group in array analysis

**Supplemental table 2(i).** Genes which were upregulated after lipopolysaccharides (LPS) injection in array analysis

**Supplemental table 2(j).** Genes which were downregulated after lipopolysaccharides (LPS) injection in array analysis

**Supplemental table 3.** The qRT-PCR results after general anaesthesia (S=sevoflurane, P=propofol) with or without lipopolysaccharides (LPS) injection relation to the control group (NC)

**Abbreviations for above tables:**

NC: naïve control; S: sevoflurane; P: propofol; LPS: lipopolysaccharides; CDH11: Cadherin 11; EPHB2: EPH receptor B2; EWSR1: Ewing sarcoma breakpoint region 1; FN1: Fibronectin 1; FXYD5: FXYD domain containing ion transport regulator 5; GNRH1: Gonadotropin-releasing hormone 1; HGF: Hepatocyte growth factor; HPSE: Heparanase; HRAS: V-Ha-ras Harvey rat sarcoma viral oncogene homolog; HTATIP2: HIV-1 Tat interactive protein 2; IGF1; Insulin-like growth factor 1; IL18: Interleukin 18; IL1β: Interleukin 1beta; ITGA7: Integrin, alpha7; ITGB3: Integrin beta 3; KISS1: KiSS-1 metastasis-suppressor; KISS1R: KISS1 receptor; KRAS: V-Ki-ras2 Kirsten rat sarcoma viral oncogene homolog; MCAM: Melanoma cell adhesion molecule; MDM2:Mdm2 p53 binding protein homolog; MET: Met proto-oncogene; METAP2: Methionyl aminopeptidase 2; MGAT5: Mannosyl (alpha-1,6-)-glycoprotein beta-1,6-N-acetyl-glucosaminyltransferase; MMP10: Matrix metallopeptidase 10; MMP11: Matrix metallopeptidase 11; MMP13: Matrix metallopeptidase 13; MMP2: Matrix metallopeptidase 2; MMP3: Matrix metallopeptidase 3; MMP7: Matrix metallopeptidase 7; MMP9: Matrix metallopeptidase 9; MTA1: Metastasis associated 1; MTSS1: Metastasis suppressor 1; MYC: V-myc myelocytomatosis viral oncogene homolog; MYCL1: V-myc myelocytomatosis viral oncogene homolog 1; NF2: Neurofibromin 2; NME1: Non-metastatic cells 1; NME4: Non-metastatic cells 4; NR4A3: Nuclear receptor subfamily 4, group A, member 3; PLAUR: Plasminogen activator, urokinase receptor; PNN: Pinin, desmosome associated protein; PTEN: Phosphatase and tensin homolog; RORB: RAR-related orphan receptor B; RPSA: Ribosomal protein SA; SERPINE1: Serpin peptidase inhibitor; clade E: nexin, plasminogen activator inhibitor type 1), member 1; SET: SET nuclear oncogene; SMAD2: SMAD family member 2; SMAD4: SMAD family member 4; SRC: V-src sarcoma (Schmidt-Ruppin A-2) viral oncogene homolog; SYK: Spleen tyrosine kinase; TIMP2: TIMP metallopeptidase inhibitor 2; VEGFA: Vascular endothelial growth factor A.


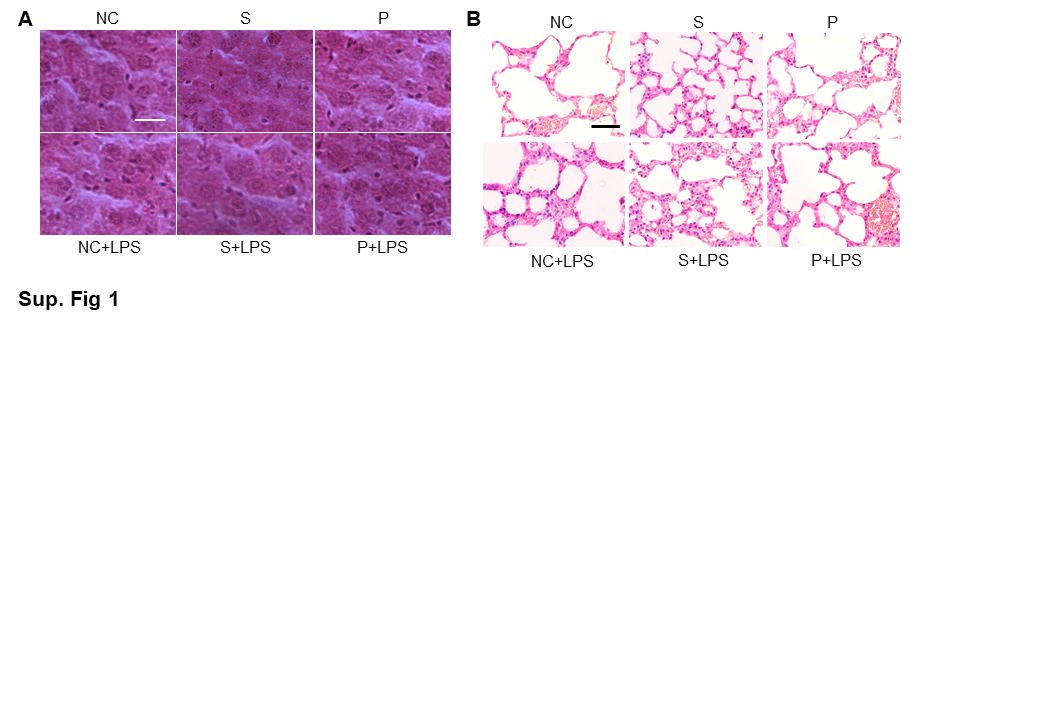


**Supplementary figure 1**: The liver and lung H and E staining sections **1A**: H and E staining images from paraffin fixed liver sections at Day 28. x20 magnification, scale bar=20μm. **1B**. H and E staining images from paraffin fixed lung sections at Day 28. x20 magnification, scale bar=20μm. NC: naïve control, S: sevoflurane, P: propofol, LPS: lipopolysaccharides.
